# Postpartum Weight Retention and Its Determinants in Lebanon and Qatar: Results of the Mother and Infant Nutrition Assessment (MINA) Cohort

**DOI:** 10.3390/ijerph17217851

**Published:** 2020-10-27

**Authors:** Lara Nasreddine, Jennifer Ayoub, Nada Abbas, Mariam Abdul Malik, Farah Naja

**Affiliations:** 1 Nutrition and Food Sciences Department, Faculty of Agriculture and Food Sciences, American University of Beirut, Beirut 110236, Lebanon; ln10@aub.edu.lb (L.N.); ja88@aub.edu.lb (J.A.); abbas_nada@hotmail.com (N.A.); 2 Primary Health Care Corporation, Doha 26555, Qatar; mabdulmalik@phcc.gov.qa

**Keywords:** postpartum weight retention, determinants, diet, cohort, Lebanon, Qatar

## Abstract

Excessive Postpartum Weight Retention (PWR) is postulated to increase the risk of adverse health outcomes for mothers and offspring. Using data from the Mother and Infant Nutritional Assessment (MINA) cohort in Lebanon and Qatar, this study aimed to examine PWR and its determinants at 6 months after delivery. Pregnant women (*n* = 183) were recruited during their first trimester and were followed up through pregnancy and after delivery. During this period, face-to-face interviews as well as extraction from medical charts were conducted to collect data regarding the socioeconomic, anthropometric and dietary intake of participants. The mean PWR (kg) among participants was 3.1 ± 5.6 at delivery, and 3.3 ± 5.3 and 2.7 ± 4.7 at 4 and 6 months after delivery, respectively. Results of the multiple logistic regression analyses showed that a Qatari nationality and excessive GWG were associated with higher odds of a high PWR (above median) while an insufficient GWG had lower odds. After adjustment for energy, participants with a high PWR reported a greater intake of proteins, Trans fat, cholesterol, sodium and lower intakes of mono and polyunsaturated fat as compared to those with a low PWR (below median). These findings suggested priority areas for interventions to prevent excessive PWR amongst women of childbearing age in Lebanon and Qatar.

## 1. Introduction

Pregnancy is a biological process that induces physiological changes in body composition and predisposes women to significant weight gain during the childbearing years [1]. The postpartum months following childbirth have also been identified as a critical window for excess weight retention and weight cycling in women of reproductive age [2]. Postpartum weight retention (PWR) is defined as the difference between body weight at a specific time after delivery and weight prior to pregnancy [3]. Previous studies have shown that average weight retention ranges between 1.5 and 5 kg at 6–12 months postpartum [4,5], with substantial variability between women [6,7,8]. Importantly, PWR may affect the long term weight gain trajectory in women of childbearing age, increasing the risk of lifetime overweight and obesity [1,9,10]. In comparison with weight gain during other periods of life, excess weight retention after childbirth may be particularly harmful, given that it is preferentially deposited in central rather than peripheral sites [1,11]. High PWR was suggested to increase the risk of adverse maternal health outcomes, including insulin resistance, metabolic syndrome and cardiovascular diseases [12,13] and to exert harmful health outcomes on the offspring, contributing to the inter-generational cycle of obesity and associated non-communicable diseases (NCDs) [14,15].

The potential adverse health impacts of excessive PWR highlights the importance of identifying modifiable risk factors for weight retention after childbirth [16]. Available evidence suggests that excess gestational weight gain (GWG) [6,17] is an important risk factor for greater PWR, while varied and discordant findings were reported for other risk factors such as parity [18,19], maternal age [20], pre-pregnancy BMI [21,22,23] and smoking cessation [24]. Few studies have examined the association between diet, physical activity and postpartum weight change [24,25]. High caloric intake and insufficient physical activity were found to be positively associated with excessive PWR in some studies [26], but not in all [27]. High trans fat intake [3,25] and high dietary glycemic load [28] were also suggested as potential risk factors for excessive PWR. Gaining a greater insight into the possible determinants of PWR would enable the development of more targeted preventive strategies and behavior change interventions [29].

Most studies of PWR have focused on women living in Western or Asian countries, and hence findings may not be easily extrapolated to other contexts and settings [16] such as the Eastern Mediterranean region (EMR) [16,24,29,30,31,32,33,34]. Countries of the EMR are witnessing the nutrition transition with its characteristic shifts in diet, lifestyle and a disquieting escalation in the prevalence of nutrition-related NCDs [35]. The EMR also harbors one of the highest rates of overweight and obesity among women of childbearing age worldwide, with estimates reaching as high as 79% in some instances [36,37,38]. Despite this high burden of obesity in women, and despite the recognition of PWR as an important risk factor for lifetime obesity [39], research investigating the magnitude and determinants of PWR are completely lacking in countries of the region. To move this agenda forward, a collaborative research endeavor was conducted between Lebanon and Qatar to launch the first mother and child cohort in the EMR, which investigates the impact of maternal nutritional status and lifestyle on neonatal outcomes [40], and examines the association of nutrition imbalances early in life with birth outcomes, growth patterns and early determinants of NCDs [40]. The “Mother and Infant Nutritional Assessment” (MINA) cohort, is a three year follow-up study of pregnant women and their children, which was initiated in 2015 in two Arab countries of the EMR; the first represents middle-income fossil fuel-importer countries (Lebanon) and the second represents high income fossil fuel exporters (Qatar) [40,41]. Using data stemming from the MINA cohort, the objective of this study is to characterize PWR in Lebanon and Qatar, and examine socioeconomic, anthropometric and dietary determinants of PWR at six months after delivery. The identification of factors associated with weight retention among postpartum women can be the basis for the development of primary public health strategies to curb the obesity epidemic among women in the EMR.

## 2. Materials and Methods

### 2.1. Study Design and Subjects

The MINA cohort is a three-year follow-up study targeting pregnant women and their children, residing in Beirut, Lebanon and Doha, Qatar. Pregnant women in their first trimester (0–13 weeks of gestation) were recruited from primary healthcare centers and private clinics, between November 2015 and December 2018. In Lebanon, data were collected from the two largest private and governmental hospitals and from 6 primary health centers. In Qatar, a total of 10 primary health centers were visited for recruitment. Inclusion criteria were being of Lebanese nationality (for the Lebanese arm of the cohort), Qatari nationality, or non-Qatari residing in Qatar for a minimum of 5 years (for the Qatari arm of the cohort), pregnant with a singleton and absence of chronic illnesses that may affect dietary intake. Throughout the MINA cohort follow up, study participants were interviewed for data collection during 9 visits. The first 3 visits took place at each trimester during pregnancy and were conducted at the healthcare facility. The remaining 6 visits were conducted at the participants’ homes at 4 months, 6 months, 9 months, 12 months, 18 months and 24 months postpartum. In addition to these visits, delivery data were retrieved from the medical records at the hospitals. This data included information related to maternal weight before and after delivery, complications during pregnancy and during delivery and birth outcomes. The detailed protocol of the MINA study has been described elsewhere [40]. For the purpose of this paper, data were used from 3 visits (visit 1 (first trimester at the healthcare facility), visit 4 (4 months postpartum home visit) and visit 5 (6 months postpartum home visit)), in addition to data retrieved from the hospital medical records. The study was approved by the Institutional Review Board at the American University of Beirut in Lebanon (Protocol ID: NUT.FN.12) and by the Primary Health Care Corporation in Qatar (Protocol ID: PHCC/RC/15/04/006). Written informed consent was obtained from all participants prior to enrollment in the study.

### 2.2. Data Collection

Trained field workers approached potential study participants at the healthcare facility’s waiting room. The study aims and protocol were explained, and a written consent was obtained from interested and eligible participants prior to the face-to-face interview. During this visit (visit 1), participants completed a multi-component sociodemographic, lifestyle and dietary questionnaire. After completing the questionnaire, the height and weight of the participant were measured. Collected sociodemographic characteristics included age (in years), country of residence (Lebanon, Qatar), participant’s employment status (housewife, employed), participant’s education level (up to high school, university), income (low (<$1000), medium ($1000–$2000) and high (>$2000)), and number of children (0, ≥1; excluding the current pregnancy). The lifestyle section included questions about smoking (non-smoker, smoker), pre-pregnancy breakfast consumption (regular (three or more days per week), non-regular (less than three days per week)), and physical activity during pregnancy (low, moderate, high). Information regarding physical activity was collected using the Pregnancy Physical Activity Questionnaire (PPAQ). Total physical activity was defined in MET-minutes (multiples of the resting metabolic rate for an activity multiplied by the minutes performed) and was calculated by weighting each type of activity by its energy requirements. Physical activity was categorized into low, moderate and high intensity, assigned to the 1st, 2nd and 3rd tertiles of METS-min per week. Height was measured to the nearest 0.1 cm based on standard protocol using the Seca 213 stadiometer, and weight was measured to the nearest 0.1 kg using the Seca 877 scale. Pre-pregnancy weight (kg) was reported by the participants. During this visit, pre-pregnancy weight was reported by the participants, in kg. Pre-pregnancy BMI was calculated as the respective weight (in kg) divided by the square of height (in m^2^) and stratified according to the WHO criteria [42].

During visit 1, information regarding usual dietary intake before pregnancy was collected using a culture-specific 98-item semi-quantitative food frequency questionnaire (FFQ). Food items were categorized into 24 groups and were composed of single items and composite traditional recipes. Subjects were asked to indicate the number of times each food item was eaten per day, per week, per month or never, and the average amount eaten per food item, in grams or in comparison to reference portion sizes listed for each item. To assist in portion-size determination, participants were provided with a two-dimensional visual aid [43] and pictures of common household measures, measuring cups and spoons. The indicated frequency of each consumed food and beverage was converted to daily intake. The food composition database of the Nutritionist Pro™ software (Axxya Systems LLC, Stafford, TX, USA) was then used to compute the intake of energy, macronutrients and micronutrients. An open-ended section was also included at the end of the FFQ for the purpose of reporting additional foods and beverages consumed at least weekly.

From the hospital medical records, weight (kg) when admitted for delivery at the hospital was extracted. This variable was used to compute the GWG (kg), after subtracting from the weight collected at visit 1. GWG was then categorized based on the 2009 Institute of Medicine recommendations as insufficient (<12.5 kg if underweight, <11.5 kg if normal weight, <7 kg if overweight and <5 kg if obese), adequate (12.5–18 kg if underweight, 11.5–16 Kg if normal weight, 7–11.5 kg if overweight and 5–9 kg if obese) and excessive (>18 kg if underweight, >16 kg if normal weight, >11.5 kg if overweight and >9 kg if obese) [44]. Moreover, from the medical charts, the weight post-delivery (kg) was extracted. In addition, the following information was also extracted from the medical records (type of delivery (Caesarean vs. normal/vaginal); occurrence of delivery complications (no vs. yes); delivery of preterm vs. full term baby).

**Figure 1:**
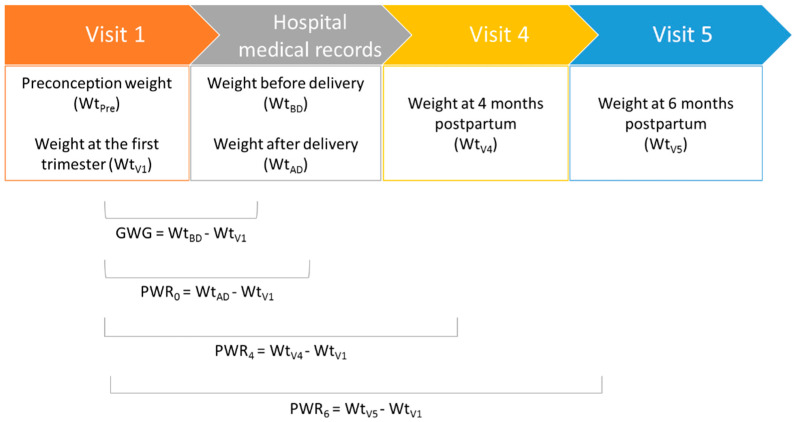
Timeline of anthropometric data collection.

During visit 4 (4 months postpartum) and visit 5 (6 months postpartum), both of which took place at the participants’ home, the weight of the mother was measured following the same protocol used during visit 1. PWR at 4 months postpartum (PWR_4_) (kg) was calculated as the difference between the subject’s weight collected at visit 4 (4 months postpartum home visit) and that collected during visit 1 (at the healthcare facility’s private collection area). Similarly, PWR at 6 months postpartum (PWR_6_) (kg) was calculated as the difference between the weight collected at visit 5 (6 months postpartum home visit) and that collected at visit 1. Figure 1 summarizes the timeline for the collection of anthropometric data. Information with regards to exclusive breastfeeding was also collected during visit 5 (6 months postpartum).

### 2.3. Statistical Analysis

Data analysis were conducted using IBM SPSS statistics software version 25 for Windows. Descriptive statistics were presented as frequency and percentage for categorical variables and mean ± standard error (SE) for continuous variables. The main outcome, PWR_6_, was dichotomized based on the 50th percentile of the distribution. Comparisons between below versus above PWR_6_’s median were done using chi-square test and independent sample t-test for categorical and continuous variables, respectively. Simple and multiple logistic regression analyses were conducted to describe the association between the main outcome (PWR_6_ >2.4 kg vs. ≤2.4 kg) and independent variables (sociodemographic and lifestyle variables). In the multiple logistic regression model, in addition to age, variables with a *p*-value < 0.2 at the univariate level were entered in the model including country, number of children, GWG, smoking and mother’s education. Results were expressed as odds ratios (ORs) with the corresponding 95% confidence interval (CI). Differences in macro- and micro- nutrients intake between both PWR_6_ groups and countries (Lebanon and Qatar) were examined using independent sample *t*-test. A two-way ANOVA was also conducted in order to test for an interaction between PWR_6_ and country with the macro- and micro-nutrients as outcomes (for these comparisons, the macro- and micro-nutrient intakes were adjusted for energy using the residual method) [45]. *p*-values lower than 0.05 were considered statistically significant.

## 3. Results

**Figure 2:**
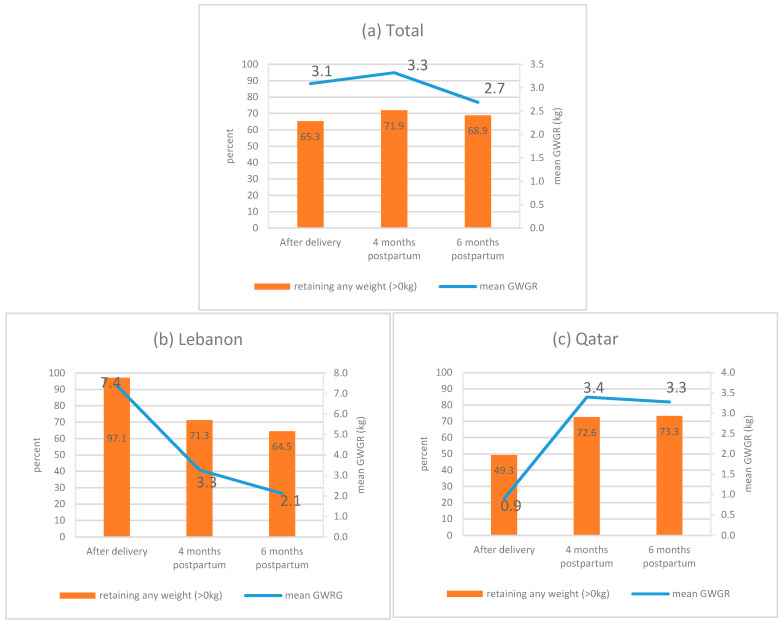
Mean PWR and percentage of women retaining any weight (>0 kg) at PWR_0_, PWR_4_, and PWR_6_ among: (**a**) the total sample; (**b**) among Lebanese residents; and (**c**) among Qatari residents. Abbreviation: mean PWR: mean postpartum weight retention; PWR_0_: postpartum weight retention after delivery; PWR_4_: postpartum weight retention at 4 months; PWR_6_: postpartum weight retention at 6 months.

Figure 2 represents the mean PWR and percentage of women retaining any weight (>0 kg) at PWR_0_, PWR_4_ and PWR_6_ among (a) the total sample, (b) Lebanese residents and (c) Qatari residents. In the total sample (Figure 2a), a slight increase in mean PWR was shown between the after delivery time period and 4 months postpartum, followed by a decrease at 6 months postpartum (mean PWR: 3.1, 3.3 and 2.7 kg, respectively). Similar trends were observed in the probability of retaining any weight (>0 kg) (65.3%, 71.9% and 68.9%, respectively). When stratified by country of residence (Figure 2b,c), opposing directions in the trend of PWR indicators were observed between the after-delivery time periods and at four months postpartum. Among Lebanese residents, mean PWR decreased from 7.4 kg to 3.3 kg and the probability of retaining any weight (>0 kg) decreased from 97.1% to 71.3%. In Qatar, an increasing trend in the PWR indicators was observed after delivery and at four months postpartum (mean PWR from 0.9 kg to 3.4 kg and the probability of retaining any weight (>0 kg) from 49.3% and 72.6%). When comparing data at four months postpartum and six months postpartum, PWR indicators decreased for both Lebanese and Qatari residents, with the former having lower PWR values at six months postpartum (mean PWR: 2.1 kg vs. 3.3 kg and probability of retaining any weight (>0 kg): 64.5% vs. 73.3%; for Lebanese and Qatari residents, respectively). It remains important to note that, among study participants, sizeable proportions had postpartum weight loss (34.7% at delivery, 28.1% at 4 months and 31.1% at 6 months).

Table 1 describes the sociodemographic, lifestyle and pregnancy characteristics of the study participants in the total sample stratified by PWR_6_ (below median vs. above median). In the total sample, mean PWR_6_ was 2.69 ± 0.35 kg, with a median of 2.4 kg. Mean maternal age was 28.04 ± 0.37 years, where 25.7% were younger than 25 years and 38% were 30 years or older. The sample was almost equally distributed across countries, with 50.8% and 49.2% from Lebanon and Qatar, respectively. Almost half of the participants (46.9%) were employed, with the majority (85%) having a university degree or higher. The majority of participants belonged to either the medium or high income categories with only 10.9% reporting an income below $1000. One third (30.4%) of the participants were primiparous. The percentage of women having a BMI < 25 kg/m^2^ was 55.7% at pre-pregnancy and 52.5% during their first trimester. As for GWG, 32% gained adequate weight during their pregnancy, 36.6% gained insufficient weight and 31.4% gained excessive weight. Around 70% of participants had a normal delivery and 55.8 had no complications during delivery. The majority of newborns (92.7%) were full-term babies. Exclusive breastfeeding was reported by 20% only of the study sample. More than two thirds (78.7%) of the sample were non-smokers prior to their pregnancy, and 77.1% reported having breakfast on a regular basis (Table 1).

When comparing participants based on the PWR_6_ median cut-off, 92 women (50.3%) had a PWR_6_ falling below the median and 91 (49.7%) above the median, with mean PWR_6_ being of −0.92 ± 0.25 kg and 6.34 ± 0.37 kg, respectively (*p*-value < 0.001). Mean age was similar across both PWR_6_ groups (28.34 ± 0.54 and 27.74 ± 0.5 years for those below and above PWR median groups, respectively) (*p*-value 0.411). Country of residence and GWG were significantly associated with PWR_6_, whereby, compared to women having a PWR_6_ below the median, those with high PWR_6_ were more likely to be Qatari residents (58.2% vs. 40.2%, *p*-value 0.015) and to have excessive GWG (44.3% vs. 18.4%, *p*-value < 0.001). Results also showed that the proportion of women having 1 or more children was higher in the below median group (75.9%) as compared to those in the above-median group (63.5%); however, the difference did not reach statistical significance (*p*-value 0.081). Moreover, the proportions of women having an education up to high school level, as well as women smoking before pregnancy were both higher in the below-median PWR_6_ group, but did not reach statistical significance (*p*-values 0.191 and 0.113, respectively). Employment status, income, pre-pregnancy breakfast consumption and physical activity were equally distributed between the two PWR_6_ groups (*p*-values 0.936, 0.466, 0.622 and 0.364, respectively). In addition, neither pre-pregnancy BMI nor BMI at the first trimester were statistically different across the PWR_6_ groups (*p*-values 0.458 and 0.413, respectively). Comparisons of the baseline characteristics between Lebanese and Qatari participants were presented in Table S1. Overall, compared to Qatari, a higher proportion of Lebanese participants were employed (64.4% vs. 28.7%), had a Caesarian delivery (42.7% vs. 19.9%) and smoked before pregnancy (39.8% vs. 2.2%). On the other hand, Qatari participants had a higher prevalence of overweight and obesity before pregnancy (57.6% vs. 31.9%) and during the first trimester of pregnancy (62.5% vs. 33%) as compared with Lebanese participants. No significant differences were observed for the remaining baseline characteristics between Lebanese and Qatari participants. (Table S1). 

Table 2 summarizes the simple and multiple regression analyses between PWR_6_ (below vs. above median) as outcome and sociodemographic and lifestyle variable predictors. The ORs of belonging to the group of women with PWR_6_ above the median vs. below the median, as well as their corresponding 95% Cis, are presented. Model 1 depicted the crude association, and Model 2 was adjusted for potential confounders including maternal age, country of residence, number of children, GWG, exclusive breastfeeding, pre-pregnancy smoking status and education status (those with a *p*-value < 0.2 at the crude/bivariate level). In Model 1, the country of residence and GWG were significantly associated with PWR_6_. These associations remained even after adjustments in Model 2. At the crude level, the odds of having a PWR_6_ above 2.4 kg (vs below) among women residing in Qatar was 2.17 times higher than that of women residing in Lebanon (OR: 2.17; 95% CI: 1.2–3.92). This OR increased to 3.02 after adjusting for potential confounders in Model 2 (OR: 3.02; 95% CI: 1.22–7.52). Gaining insufficient weight during pregnancy was inversely associated with a higher PWR_6_, when compared to gaining adequate weight in both Model 1 (OR: 0.32; 95% CI: 0.15–0.67) and Model 2 (OR: 0.27; 95% CI: 0.1–0.69). While excessive GWG was not statistically significantly associated with PWR_6_ when compared to adequate GWG in the crude model (OR: 2.15; 95% CI: 0.97–4.76), after adjustment, a positive statistical significance association was observed (OR: 3.5; 95% CI: 1.24–9.85). Simple logistic regression analyses for the determinants of PWR_6_ conducted for each country separately showed that, in Lebanon, women with insufficient GWG had lower odds of excessive PWR_6_ (OR: 0.18; 95% CI: 0.05–0.65). In Qatar, excessive GWG was associated with a PWR_6_ above the median (OR: 6.6; 95%CI: 1.25–34.95). The small sample size limited the possibility of conducting multiple regressions for each country separately. 

Table 3 describes the results of the two way ANOVA for the associations of PWR_6_ and country (and their interactions) with energy (Kcal), macronutrients (expressed as percent contribution to total energy), and micronutrients (expressed as mg/g per 1000 kcal). Data were expressed as mean ± SE, and comparisons were tested adjusting for energy (residual method). In the total sample, mean energy consumption was 2853.35 ± 166.99 Kcal. When comparing between PWR_6_ groups, although not statistically significant (*p*-value 0.054), the mean energy consumption was shown to be higher among the group having PWR_6_ > 2.4 kg (3172.29 ± 295.74 Kcal vs. 2530.86 ± 295.74 Kcal, in the above vs. below median PWR_6_ groups respectively). As for the intake of carbohydrates, the mean percent contribution to total energy intake was shown to be 45.11 ± 0.62% Kcal in the total sample and was similar across PWR_6_ groups (*p*-value 0.256). The percent contribution of protein intake to total energy intake was significantly higher in the above-median PWR_6_ group (15.98 ± 0.45% Kcal vs. 15.2 ± 0.42% Kcal, *p*-value 0.026), whereas that of fat intake was significantly higher in the below-median PWR_6_ group (40.12 ± 1.06% Kcal vs. 38.7 ± 0.63% Kcal, *p*-value 0.037). The percent contribution of both monounsaturated and polyunsaturated fat was significantly higher in the below-median group (13.99 ± 0.46% Kcal vs. 13.42 ± 0.29% Kcal for monounsaturated fats, and 10.89 ± 0.56% Kcal vs. 10.23 ± 0.31% Kcal for polyunsaturated fats, *p*-values 0.024 and 0.039, respectively), whereas that of trans fatty acid was significantly lower (0.22 ± 0.02% Kcal vs. 0.27 ± 0.02% Kcal, *p*-values 0.025). Energy-adjusted cholesterol and sodium intake were also statistically significantly lower in the below median PWR_6_ group (125 ± 6.15 mg/1000 Kcal vs. 131.07 ± 5.4 mg/1000 kcal for cholesterol; 980.12 ± 25.26 mg/1000 kcal vs. 1018.69 ± 25.11 mg/1000 kcal for sodium) (*p*-values 0.049 and 0.028, respectively). The percent contribution of saturated fat and sugar to total energy intake among the total sample was not statistically significantly associated with PWR_6_ (11.6 ± 0.21% Kcal, *p*-value 0.405; 15.54 ± 0.47% Kcal, *p*-value 0.242, respectively). Energy-adjusted calcium intake was similar between the two comparison groups (403.74 ± 14.02 mg/1000 Kcal vs. 417.41 ± 15.63 mg/1000 Kcal in the below median and above median groups, respectively; *p*-value 0.11). Iron and dietary fiber intake per 1000 kcal did not reach statistical significance, although results were shown to be slightly higher in the below-median PWR_6_ group (*p*-values 0.547 and 0.878). Comparisons across the country showed that Qatari women, compared to Lebanese women, consumed more proteins (17.08% ± 0.5 vs. 14.19% ± 0.32; *p* < 0.001), less total, poly- and monounsaturated fat (37.16% ± 0.76 vs. 41.54% ± 0.91; *p* < 0.001; 12.33 ± 0.28 vs. 10.82 ± 0.31; *p* = 0.001 and 14.81 ± 0.4 vs. 12.52 ± 0.32; *p* < 0.001) and more cholesterol (143.87 ± 6.11 vs. 113.09 ± 5.01; *p* < 0.001). Except for energy, no significant interactions between PWR_6_ and country were observed (Table 3). For energy, among Qatari participants, energy was statistically significantly higher in the above median PWR_6_ group (3852.12 ± 479.54 vs. 2612.04 ± 150.73, *p*-value 0.043) while no significant association was detected among Lebanese participants (2479.2 ± 221.88 vs. 2224.12 ± 130.36 for below and above median PWR_6_ respectively, *p*-value 0.379) The absolute intake of macro- and micronutrients in association with PWR_6_ are presented in Supplementary Table S2.

## 4. Discussion

This study is the first from the EMR to characterize PWR and to identify factors that may increase the risk of excessive weight retention at 6 months postpartum in women of childbearing age. The study showed that average PWR at 6 months was of 2.1 kg and 3.3 kg among Lebanese and Qatari women, respectively. High PWR was found to be associated with excessive GWG and with Qatar as a country of residence. Higher dietary intakes of trans fat, cholesterol, sodium and protein were positively associated with PWR, while lower intakes of monounsaturated fatty acids (MUFAs) and polyunsaturated fatty acids (PUFAs) were observed in women who experienced excessive PWR. 

Our study showed that at six months postpartum, 68.9% of our cohort participants still retained some weight, a finding that is similar to that reported by Hollis et al., in a prospective cohort study among women in the UK [29] and Lyu et al. (2009) in a longitudinal follow up study among women in Taiwan [30]. Interestingly, different PWR trajectories were observed for women in Lebanon compared to Qatar. While between 4 and 6 months post-delivery, average PWR followed a decreasing trajectory in both countries, opposing trajectories were observed during the first 4 months postpartum: mean PWR decreased in Lebanon from 7.4 kg (PWR0) to 3.3 kg (PWR4), whereas in Qatar, mean PWR increased from 0.9 kg to 3.4 kg. The observed lower average PWR in Qatari women immediately after delivery (PWR0) may be explained by the fact that a higher proportion of Qatari women had insufficient GWG compared to Lebanese women (33.3% vs. 21.3%) [41]. The fact that different PWR trajectories were observed in our study is in line with previous reports in the literature. A longitudinal cohort in China has documented distinct PWR trajectories among women [31], with class 3 trajectory being characterized by a consistent slight decreasing trend in weight during the first 8 months post-delivery, while class 2 trajectory was characterized by a rapid increase in PWR [31]. Among the factors that explained the increasing trajectory in PWR were maternal obesity [31]. In our cohort, and as shown in our previous study [41], the prevalence of high pre-pregnancy BMI was significantly higher in Qatar (58%) compared to Lebanon (30.8%), and the Qatari nationality was found to be an independent risk factor for pre-pregnancy overweight [41]. 

There is a lack of international consensus on how to define high weight retention. In this study, high PWR was defined as weight retention exceeding the median value of 2.4 kg at 6 months postpartum. The observed average PWR at 6 months postpartum (2.69 kg for the total population) is lower than that reported from the US (5.6 kg) [34] and Brazil (4.8 kg) [24], while being in the range of values reported from Asian countries such as Taiwan, China and Malaysia (2.1–3.25 kg) [16,30,31,32,33]. Excess PWR may increase the risk of lifetime obesity in women of childbearing age. In a cohort of US women, Rooney and Schauberger [46] have investigated the effect of PWR and long-term weight changes a decade after pregnancy. Their findings showed that women who lost the weight gained during pregnancy were more likely to have a lower follow-up BMI compared to those who retained weight at 6 months postpartum [46]. Of concern is the fact that PWR may be physiologically more harmful than weight gain acquired at other times in life [3]. Excess weight retention after pregnancy is preferentially deposited centrally [1,11], and in turn central adiposity is closely linked to insulin resistance and increased cardiovascular disease risk [47,48]. A recent study based on the VIVA project in the US showed that women who retained weight during the first 2 y postpartum developed an adverse cardiometabolic profile 3 years after delivery [49], characterized by a higher waist circumference, low-density lipoprotein cholesterol and inflammatory markers. 

In our study, the odds of having high PWR were three times higher among women living in Qatar compared to those living in Lebanon. This finding is potentially reflective of the higher overall prevalence of overweight and obesity in Qatar (and other Gulf Cooperation Council (GCC) countries) [50,51,52], compared to Lebanon (and the Levant area) [36,53,54]. Qatar is in fact classified as a country in advanced nutrition transition stage, characterized by alarming surges in the levels of overweight and obesity and substantial shifts in diet and lifestyle towards westernized patterns, while Lebanon is still classified as a country in early nutritional transition stages, with relatively moderate levels of overweight and obesity [55]. Our findings also showed that excessive GWG was a significant predictor of high PWR at six months postpartum. This is in line with previous studies conducted in Asian, European and American populations, indicating that excessive GWG may be the single most important factor determining PWR [24,31,32,33,34,56]. In our cohort, almost a third of women had excessive GWG when compared to the recommendations of the Institute of Medicine, highlighting the magnitude of the problem. The observed association between PWR and excessive GWG underscores the importance of promoting adequate weight gain during pregnancy and the appropriateness of the Institute of Medicine GWG guidelines to minimize PWR in childbearing women [46]. However, GWG is not regularly monitored in antenatal care, and its potential implications on maternal and child health risk are often neglected [29,41,57]. There have been repetitive calls to formally integrate weight monitoring and management into routine antenatal care through the development of practice-level policies [29,57]. Health practitioners, especially obstetricians, have frequent contact with women during the pregnancy and are thus placed in an ideal position to support women to manage GWG [58] or refer them to dietitians when needed [29]. 

In our study, caloric intake was higher in women with high PWR compared to those with lower PWR (3172 vs. 2531 kcal/d). However, this difference did not reach statistical significance, possible due to the small sample size of the cohort. Despite the scarcity of studies investigating diet as a determinant of PWR, available evidence suggests that higher energy intake may predict excessive PWR [16,30]. In their multivariable analysis, Lyu et al. [30] reported that energy intake could explain 24% of the variation in weight retention at 6 months postpartum, and suggested the reduction in dietary energy intake as a strategy to prevent unhealthy weight retention and obesity after delivery. Interestingly, in our study, trans fat intake was found to be an independent predictor of higher PWR among women at six months after delivery. Although few studies have investigated the role of nutrients’ intakes in PWR, the observed association between trans fat and PWR is in agreement with that reported by Oken et al. in a prospective cohort study of 902 women enrolled in Project Viva in the US [3]. While evidence linking intake of trans fat with adverse blood cholesterol profiles and risk of coronary heart disease is well recognized [59,60], recent studies suggest that trans fat intake may also be associated with higher body weight, weight gain and increasing waist circumference in non-pregnant adults [61,62], possibly through its role in increasing systemic inflammation [63,64,65]. Trans fat intake may also be a marker for other unhealthy dietary intakes or patterns rather than being causally associated with weight gain [3]. In our study, and besides trans fat, higher intakes of cholesterol, sodium and protein were also associated with higher PWR, coupled with lower intakes of MUFAs and PUFAs. Taken together, these findings may reflect a nutrient profile that is usually characteristic of the westernized dietary pattern that is rich in animal-based and processed food products [66]. Previous studies have shown that the Western dietary pattern is typically high in meat and fast food while being associated with higher intakes of energy, protein and cholesterol [67]. In Lebanon, Naja et al. showed that, compared to the traditional Lebanese pattern, the Western dietary pattern was associated with higher intakes of sodium and cholesterol [66]. Adherence to the Western pattern was also shown to increase the risk of obesity in Lebanese adults [66] and Qatari women [68]. Hence, it may not be surprising that higher weight retention was associated with a nutrient intake profile that usually characterizes the Western dietary pattern. 

Our study did not show any significant association between pre-pregnancy BMI and PWR at six months post-partum. Conflicting findings are in fact reported in the literature with respect to the relation between pre-gravid BMI and PWR [4,21,32,69,70]. In agreement with our findings, Shao et al. (2018) and Lyu et al. (2009) did not find a significant relationship between pre-gravid BMI and PWR at six months post-partum among women from Taiwan [30,32]. In contrast, higher pre-pregnancy BMI was suggested as a determinant of PWR in a number of studies conducted in the US and the UK [69,71,72], while opposite findings were reported by Krause et al. (2010) in the US, whereby increased pre-pregnancy BMI was negatively associated with PWR [34]. This discrepancy in findings highlights the need for further explorations on the impact of pre-pregnancy BMI on PWR in various populations [32]. Similarly, exclusive breastfeeding for six months was not associated with PWR in Lebanese and Qatari women. Although breastfeeding increases the daily energy expenditure of lactating women, there have been inconsistent results on the association between breastfeeding and PWR, with some studies reporting a modest effect on weight loss [34,73] and others revealing a small or no effect [33,74]. A recent systematic review of prospective and retrospective observational studies reported little or no association between breastfeeding and weight loss or change in body composition [74]. The multifactorial nature of weight retention or weight loss, and the contextual factors associated with breastfeeding imply that the association may not be generalizable to all women [74]. 

The strengths of this study comprise its prospective nature, which allows an exploration of causal relationships whilst requiring less recall compared to other epidemiological study designs [75,76]. Furthermore, although the MINA cohort is a multi-country cohort, the study protocols and procedures were standardized across both data collection sites. Weight retention was assessed based on actual measurements of weight at different points in time, rather than relying on self-reported weight by study participants. For the calculation of PWR, and as suggested by the Institute of Medicine [77], women’s weight post-delivery was compared to weight measured during the first prenatal visit (first trimester), which corresponded to 4–6 weeks of gestation. 

However, the results of this study ought to be considered in light of the following limitations. First, the small sample size in our study may have resulted in underpowered analyses. Second, data pertinent to pre-pregnancy BMI and GWG were collected from the participants’ medical records. Although standards techniques were adopted by the clinics and health care centers for the measurement of body weight, the possibility of random errors in these measurements cannot be ruled out [41]. Third, in our study, and similarly to the study by Hollis et al. (2017) in the UK, pre-pregnancy diet was included as a proxy of postpartum diet [29]. However, previous studies have documented a high correlation between diets during early pregnancy and after childbirth [30]. Fourth, socio-demographic and lifestyle characteristics were assessed using a questionnaire that was administered in an interview setting. As observed in most questionnaire-based studies, the interview-based approach may lead to social desirability bias [78]. In our study, fieldworkers had received extensive training before the initiation of data collection in order to decrease judgmental verbal and nonverbal communication and therefore minimize the likelihood of social desirability bias. 

## 5. Conclusions

In conclusion, this study showed that the majority of women in our cohort had retained their weight six months post-partum, with the average PWR being estimated at 2.69 kg. Women living in Qatar had three times higher odds of experiencing high PWR compared to those living in Lebanon, highlighting the advanced stage of the nutrition transition in Qatar compared to Lebanon. Factors that were found to be associated with higher PWR included excessive GWG, and high dietary intakes of trans fat, sodium, cholesterol and protein, which are usually reflective of the Western dietary pattern. The study findings therefore identified priority areas for intervention to prevent excessive PWR amongst women of childbearing age in two countries of the EMR. Improving medical staff education with respect to the IOM guidelines for GWG may help and support pregnant women in maintaining GWG within the adequate range according to BMI. Dietary interventions spanning the entire period from pre-pregnancy through the postpartum months are also warranted to improve nutritional intakes of women of reproductive age in countries in the region. By decreasing PWR and adiposity in women, such behavioral change interventions may contribute towards the prevention of obesity in the offspring, thus offering an opportunity to simultaneously optimize health, and curb the obesity epidemic across generations. 

## Figures and Tables

**Table 1:** Sociodemographic and lifestyle characteristics of study participants stratified by PWR_6_ (below and above median values).

Participants’ Characteristics	Total (*n* = 183)	PWR_6_ below Median (≤2.4 kg) (*n* = 92)	PWR_6_ above Median (>2.4 kg) (*n* = 91)	*p*-Value
**Maternal age (years)**				0.247
18–24.9	46 (25.7)	22 (24.7)	24 (25.7)	
25–29.9	66 (36.3)	28 (31.5)	37 (41.1)	
≥30	68 (38.0)	39 (43.8)	29 (32.2)	
**Country of residence**				**0.015**
Lebanon	93 (50.8)	55 (59.8)	38 (41.8)	
Qatar	90 (49.2)	37 (40.2)	53 (58.2)	
**Employment status**				0.936
Housewife	94 (53.1)	47 (53.4)	47 (52.8)	
Employed	83 (46.9)	41 (46.6)	42 (47.2)	
**Education**				0.191
Up to high school *	26 (15.0)	16 (18.6)	10 (11.5)	
University or higher	147 (85.0)	70 (81.4)	77 (88.5)	
**Income**				0.466
Low, <$1000	11 (10.89)	7 (12.73)	4 (8.7)	
Medium, $1000–$2000	24 (23.76)	15 (27.27)	9 (19.57)	
High, >$2000	66 (65.35)	33 (60)	33 (71.74)	
**Number of children**				0.081
0	51 (30.4)	20 (24.1)	31 (36.5)	
1 or more	117 (69.6)	63 (75.9)	54 (63.5)	
**Pre-pregnancy BMI ^¶^**				0.458
Underweight and normal (<25 kg/m^2^)	98 (55.7)	46 (52.9)	52 (58.4)	
Overweight and obese (≥25 kg/m^2^)	78 (44.3)	41 (47.1)	37 (41.6)	
**First trimester BMI ^¶^**				0.413
Underweight and normal (<25 kg/m^2^)	94 (52.5)	44 (49.4)	50 (55.6)	
Overweight and obese (≥25 kg/m^2^)	85 (47.5)	45 (50.6)	40 (44.4)	
**GWG (kg)**				**<0.001**
Insufficient	64 (36.6)	46 (52.9)	18 (20.5)	
Adequate	56 (32.0)	25 (28.7)	31 (35.2)	
Excessive	55 (31.4)	16 (18.4)	39 (44.3)	
**Type of delivery**				0.871
Caesarean	55 (30.9)	27 (30.3)	28 (31.5)	
Normal/vaginal	123 (69.1)	62 (69.7)	61 (68.5)	
**Occurrence of delivery complication**				0.113
No	92 (55.8)	53 (61.6)	39 (49.4)	
Yes	73 (44.2)	33 (38.4)	40 (50.6)	
**Preterm/full term delivery**				0.435
Full term	165 (92.7)	83 (91.2)	82 (94.3)	
Preterm	13 (7.3)	8 (8.8)	5 (5.7)	
**Exclusive Breastfeeding for 6 months**				0.302
No	30 (20.7)	13 (17.3)	17 (24.3)	
Yes	115 (79.3)	62 (82.7)	53 (75.7)	
**Pre-pregnancy smoking status**				0.113
Non-smoker	144 (78.7)	68 (73.9)	76 (83.5)	
Smoker	39 (21.3)	24 (26.1)	15 (16.5)	
**Pre-pregnancy breakfast consumption**				0.622
Regular **	138 (77.1)	68 (75.6)	70 (78.7)	
Not regular	41 (22.9)	22 (24.4)	19 (21.3)	
**Physical activity**				0.364
Low	42 (33.1)	18 (32.1)	24 (33.8)	
Moderate	43 (33.9)	16 (28.6)	27 (38.0)	
High	42 (33.1)	22 (39.3)	20 (28.2)	

Data are expressed as absolute numbers and percentages. *p*-values were derived from Chi square analyses and values in bold are statistically significant (*p*-value ≤ 0.05). * Including technical diploma. ** Three or more times per week. ^¶^ BMI was stratified according to the WHO criteria [42]. Abbreviations: PWR_6_: postpartum weight retention at 6 months; BMI: body mass index; GWG: gestational weight gain.

**Table 2:** Simple and multiple logistic regression analyses for the associations of various sociodemographic and lifestyle variables with excessive PWR_6_ (>2.4 kg).

Participants’ Characteristics	Model 1 (Crude)			Model 2 (Adjusted)		
	OR	95% CI	*p*-Value	OR	95% CI	*p*-Value
Maternal age	0.97	(0.92–1.03)	0.390	1.04	(0.94–1.14)	0.493
**Country of residence** (reference: Lebanon)						
Qatar	**2.17**	**(1.2–3.92)**	**0.010**	**3.02**	**(1.22–7.52)**	**0.017**
**Number of children** (reference: 0)						
1 or more	0.57	(0.29–1.12)	0.102	0.48	(0.18–1.24)	0.130
**GWG** (reference: Adequate)						
Insufficient	**0.32**	**(0.15–0.67)**	**0.003**	**0.27**	**(0.1–0.69)**	**0.007**
Excessive	2.15	(0.97–4.76)	0.059	**3.5**	**(1.24–9.85)**	**0.018**
**Smoking** (reference: non-smoker)						
Smoker	0.54	(0.26–1.12)	0.099	1.36	(0.45–4.12)	0.588
**Educational status** (reference: Up to High school)						
University or higher	1.81	(0.77–4.25)	0.174	2.39	(0.7–8.14)	0.165
**Occurrence of Delivery Complications** (reference: No)						
Yes	1.74	(0.94–3.24)	0.08	2.2	(0.95–5.09)	0.066
**Exclusive breastfeeding for 6 months** (reference: No)						
Yes	1.48	(0.66–3.32)	0.345			
**Preterm/full term delivery** (reference: full term)						
Preterm	0.62	(0.19–1.97)	0.415			
**Delivery type** (reference: Normal/Vaginal))						
Caesarean section	1.13	(0.6–2.14)	0.871			
**Employment status** (reference: Housewife)						
Employed	0.98	(0.54–1.77)	0.951	-	-	
**Income** (Low, <$1000)						
Medium, $1000–$2000	1.05	(0.24–4.62)	0.949	-	-	
High, >$2000	1.75	(0.47–6.62)	0.406			
**Pre-pregnancy BMI** (reference: Underweight and normal)						
Overweight and obese	0.80	(0.44–1.45)	0.459	-	-	
**First trimester BMI** (reference: Underweight and normal)						
Overweight and obese	0.82	(0.46–1.48)	0.508	-	-	
**Pre-pregnancy breakfast consumption** (reference: Regular)						
Not regular	0.82	(0.41–1.64)	0.566	-	-	
**Physical activity** (reference: Low)						
Moderate	1.20	(0.5–2.87)	0.690	-	-	
High	0.68	(0.29–1.6)	0.374	-	-	

Abbreviations: PWR_6_: postpartum weight retention at 6 months; BMI: body mass index; GWG: gestational weight gain. Model 1: Crude association; Model 2: Adjusted for maternal age, country of residence, number of children, GWG, pre-pregnancy smoking status, exclusive breastfeeding at 6 months and educational status.

**Table 3:** Energy-adjusted macro- and micronutrient intakes of study participants stratified by PWR_6_ (below and above median values) and country (Lebanon and Qatar).

Participants’ Characteristics	Total	PWR_6_			Country			
		PWR_6_ ≤ 2.4 kg	PWR_6_ > 2.4 g	*p*-Value ^i^	Lebanon	Qatar	*p*-Value ^ii^	*p*-Value ^iii^
Energy (Kcal)	2853.35 ± 166.99	2530.86 ± 147.23	3172.29 ± 295.74	0.054	2374.97 ± 141.6	3358.9 ± 300.91	0.004	0.0241
Protein (% Kcal)	15.59 ± 0.31	**15.2 ± 0.42**	**15.98 ± 0.45**	**0.026**	14.19 ± 0.32	17.08 ± 0.5	**<0.001**	0.607
Carbohydrate (% Kcal)	45.11 ± 0.62	45.15 ± 0.98	45.07 ± 0.77	0.256	45.31 ± 0.88	44.89 ± 0.89	0.085	0.141
Fat (% Kcal)	39.41 ± 0.62	**40.12 ± 1.06**	**38.7 ± 0.63**	**0.037**	41.54 ± 0.91	37.16 ± 0.76	**<0.001**	0.349
Saturated fat (% Kcal)	11.6 ± 0.21	11.62 ± 0.32	11.57 ± 0.28	0.405	12.33 ± 0.28	10.82 ± 0.31	**0.001**	0.219
Monounsaturated fat (% Kcal)	13.7 ± 0.27	**13.99 ± 0.46**	**13.42 ± 0.29**	**0.024**	14.81 ± 0.4	12.52 ± 0.32	**<0.001**	0.530
Polyunsaturated fat (% Kcal)	10.56 ± 0.32	**10.89 ± 0.56**	**10.23 ± 0.31**	**0.039**	10.58 ± 0.5	10.53 ± 0.41	0.260	0.457
Trans fatty acid (% Kcal)	0.24 ± 0.01	**0.22 ± 0.02**	**0.27 ± 0.02**	**0.025**	0.26 ± 0.02	0.22 ± 0.02	0.101	0.050
Sugar (% Kcal)	15.54 ± 0.47	15.47 ± 0.7	15.61 ± 0.63	0.242	16.74 ± 0.65	14.27 ± 0.66	0.761	0.969
Cholesterol (mg/1000 Kcal)	128.05 ± 4.08	**125 ± 6.15**	**131.07 ± 5.4**	**0.049**	113.09 ± 5.01	143.87 ± 6.11	**<0.001**	0.279
Sodium (mg/1000 Kcal)	999.51 ± 17.82	**980.12 ± 25.26**	**1018.69 ± 25.11**	**0.028**	1033.63 ± 24.76	963.45 ± 25.24	0.744	0.765
Calcium (mg/1000 Kcal)	410.62 ± 10.49	403.75 ± 14.02	417.41 ± 15.63	0.11	445.41 ± 15.51	373.85 ± 13	0.176	0.188
Iron (mg/1000 Kcal)	5.43 ± 0.13	5.54 ± 0.22	5.33 ± 0.13	0.547	5.84 ± 0.22	5 ± 0.11	0.205	0.448
Dietary fiber (g/1000 Kcal)	8.7 ± 0.22	9 ± 0.33	8.41 ± 0.3	0.878	9.49 ± 0.32	7.87 ± 0.28	0.272	0.962

Abbreviations: PWR_6_: postpartum weight retention at 6 months; Kcal: calories; mg: milligram; g: gram; %: percent. Data are expressed as mean ± SE. Numbers in bold are statistically significant (*p*-value ≤ 0.05). ^i^ *p*-value of the independent sample *t*-test comparing participants below versus above PWR_6_’s median adjusted for energy using residual method. ^ii^ *p*-value derived from the post hoc independent sample *t*-test of the two-way ANOVA comparing Lebanese and Qatari participants adjusted for energy using residual method. ^iii^ *p*-value of the interaction between PWR_6_ and country.

## Supplementary Materials

The following is available online at https://www.mdpi.com/1660-4601/17/21/7851/s1, Table S1: Sociodemographic and lifestyle characteristics of study participants stratified by Country. Table S2: Absolute macro- and micronutrients intakes of study participants stratified by PWR_6_ (below and above median values).
