# Supplementary material for: Postpartum Weight Retention and Its Determinants in Lebanon and Qatar: Results of the Mother and Infant Nutrition Assessment (MINA) Cohort
Source: Int J Environ Res Public Health. 2020 Oct 27;17(21):7851. doi: 10.3390/ijerph17217851 (PMC7672614; doi:10.3390/ijerph17217851)
Supplement: Supplementary file 1 [file ijerph-17-07851-s001.pdf]

Table 1. Sociodemographic and lifestyle characteristics of study participants stratified by Country.

	Total	Lebanon	Qatar	
	**(*n* = 183)**	**(*n* = 93)**	**(*n* = 90)**	***p*-value**
**PWR_6 (Kg)**	2.69 ± 0.35	2.12 ± 0.46	3.27 ± 0.53	0.100
				**0.015**
below median	92 (50.3)	55 (59.1)	37 (41.1)	
above median	91 (49.7)	38 (40.9)	53 (58.9)	
**Maternal age (years)**	28.04 ± 0.37	28.09 ± 0.49	28 ± 0.55	0.906
18-24.9	46 (25.7)	22 (23.9)	24 (27.6)	0.534
25-29.9	65 (36.3)	37 (40.2)	28 (32.2)	
≥ 30	68 (38)	33 (35.9)	35 (40.2)	
**Employment status**				
Housewife	94 (53.1)	32 (35.6)	62 (71.3)	**<0.001**
Employee	83 (46.9)	58 (64.4)	25 (28.7)	
**Education**				
Up to high school	26 (15)	13 (14.3)	13 (15.9)	0.773
University or higher*	147 (85)	78 (85.7)	69 (84.1)	
**Income**				
Low, <1000$	8 (12.5)	3 (8.11)	11 (10.89)	0.689
Medium, 1000-2000	16 (25)	8 (21.62)	24 (23.76)	
High, >2000$	40 (62.5)	26 (70.27)	66 (65.35)	
**Number of children**				
0	51 (30.4)	30 (37)	21 (24.1)	0.069
1 or more	117 (69.6)	51 (63)	66 (75.9)	
**Pre-pregnancy BMI ¶**				
Underweight and normal (<25kg/m²)	98 (55.7)	62 (68.1)	36 (42.4)	**0.001**
Overweight and obese (≥25kg/m²)	78 (44.3)	29 (31.9)	49 (57.6)	
**First trimester BMI ¶**				
Underweight and normal (<25kg/m²)	94 (52.5)	61 (67)	33 (37.5)	**<0.001**
Overweight and obese (≥25kg/m²)	85 (47.5)	30 (33)	55 (62.5)	
**GWG (Kg)**				
Insufficient	64 (36.6)	26 (29.2)	38 (44.2)	0.120
Adequate	56 (32)	32 (36)	24 (27.9)	
Excessive	55 (31.4)	31 (34.8)	24 (27.9)	
**Type of delivery**				
Caesarean	96 (30.1)	61 (42.7)	35 (19.9)	**<0.001**
Normal/vaginal	223 (69.9)	82 (57.3)	141 (80.1)	
**Occurrence of delivery complication**				
No	153 (56.5)	88 (61.1)	65 (51.2)	0.100
Yes	118 (43.5)	56 (38.9)	62 (48.8)	
**Preterm / full term delivery**				
Full term (≥37 weeks)	301 (93.2)	131 (92.9)	170 (93.4)	0.860
Preterm (<37 weeks)	22 (6.8)	10 (7.1)	12 (6.6)	
**Exclusive Breastfeeding for 6 months**				
No	115(79.3)	62(83.8)	53(74.6)	0.175
Yes	30(20.7)	12(16.2)	18(25.4)	
**Pre-pregnancy smoking status**				
Non-smoker	144 (78.7)	56 (60.2)	88 (97.8)	**<0.001**
Smoker	39 (21.3)	37 (39.8)	2 (2.2)	
**Pre-pregnancy breakfast consumption**				
Regular**	138 (77.1)	72 (78.3)	66 (75.9)	0.703
Not regular	41 (22.9)	20 (21.7)	21 (24.1)	
**Physical activity**				
Low	42 (33.1)	17 (30.4)	25 (35.2)	0.517
Moderate	43 (33.9)	22 (39.3)	21 (29.6)	

High	42 (33.1)	17 (30.4)	25 (35.2)

Data are expressed as mean ± SE for PWR6 and maternal age while the rest of the variable were presented as absolute numbers and percentages. Numbers in bold are statistically significant (*p*-value ≤ 0.05). * Including technical diploma. **Three or more times per week.¶ BMI was stratified according to the WHO criteria [42]. Abbreviations: PWR_6: postpartum weight retention at 6 months; BMI: body mass index; GWG: gestational weight gain.

Table 2. Absolute macro and micronutrients intakes of study participants stratified by PWR_6 (below and above median values).

	**Total**	**PWR_6 ≤2.4 (below median)**	**PWR_6 >2.4 (above median)**	***p*-value**
	**Mean ± SE**	**Mean ± SE**	**Mean ± SE**	
Energy (Kcal)	2853.35 ± 166.99	2530.86 ± 147.23	3172.29 ± 295.74	0.054
Protein (g)	111.54 ± 7.22	**92.47 ± 4.15**	**130.4 ± 13.51**	**0.008**
Carbohydrate (g)	311.1 ± 17.09	**272 ± 10.88**	**349.77 ± 31.83**	**0.023**
Fat (g)	128.88 ± 9.56	119.89 ± 13.16	137.78 ± 13.87	0.351
Saturated fat (g)	38.01 ± 2.88	34.08 ± 3.58	41.9 ± 4.48	0.175
Monounsaturated fat (g)	44.67 ± 3.26	41.3 ± 4.11	48.01 ± 5.06	0.305
Polyunsaturated fat (g)	34.85 ± 3.05	34.14 ± 5.1	35.55 ± 3.4	0.817
Trans fatty acid (g)	0.81 ± 0.08	0.67 ± 0.11	0.95 ± 0.1	0.071
Sugar (g)	102.3 ± 4.98	**90.45 ± 4.46**	**114.02 ± 8.74**	**0.018**
Cholesterol (mg)	369.9 ± 23.94	**317.01 ± 21.33**	**422.23 ± 42.12**	**0.027**
Sodium (mg)	2844.74 ± 174.89	**2459.93 ± 132.34**	**3225.36 ± 318.36**	**0.028**
Calcium (mg)	1110.9 ± 55.11	**985.29 ± 58.59**	**1235.14 ± 91.55**	**0.023**
Iron (mg)	15.03 ± 0.87	13.33 ± 0.64	16.7 ± 1.59	0.051
Dietary fiber (g)	23.31 ± 1.28	21.31 ± 0.97	25.29 ± 2.35	0.12

Abbreviations: PWR_6: gestational weight gain retention at 6 months postpartum; Kcal: calories; mg: milligram; g: gram. Data are expressed as mean ± SE. Differences among group means were tested using independent sample t-test. Numbers in **bold** are statistically significant (*p*-value ≤ 0.05).
